# Children capacity in disaster risk reduction: a call for action

**DOI:** 10.5249/jivr.v9i1.854

**Published:** 2017-01

**Authors:** Leila Mohammadinia, Davoud Khorasani-Zavareh, Ali Ardalan

**Affiliations:** ^*a*^Department of Disaster Public Health, School of Public Health, Tehran University of Medical Sciences, Tehran, Iran.; ^*b*^Department of Disaster and Emergency Health, National Institute of Health Research, Tehran University of Medical Sci-ences, Tehran, Iran.; ^*c*^Health Human Resource Research Center, School of Management and Information Sciences, Shiraz University of Med-ical Sciences, Shiraz, Iran.; ^*d*^Safety Promotion and Injury Prevention Research Center, Shahid Beheshti University of Medical Sciences, Tehran, Iran.; ^*e*^Department of Health in Disaster and Emergency, School of Health, Safety and Environment, Shahid Beheshti Universi-ty of Medical Sciences, Tehran, Iran.; ^*f*^Department of Clinical Science and Education, Södersjukhuset, Karolinska Institutet, Stockholm, Sweden.; ^*g*^Harvard Humanitarian Initiative, Harvard University, Cambridge, MA, USA.

Disasters have various physical, psychological, social and economical effects on all age groups, particularly chil-dren who are more vulnerable than adults. In the after-math of disasters, children, just like pregnant women, elderly and the disabled, are a special group with spe-cial needs. This is because they are at greater risk based on their specific physiological and psychological characteristics. Moreover, according to the Sendai docu-ment, children need to be taken more into account in Disaster Risk Reduction (DRR) program design, and poli-cy implementation should have a proactive approach.^1^

In the Sendai document, it is emphasized that policies regarding disaster risk reduction, cognition and risk perception about the risk property should be considered based upon the hazards and the environment in terms of vulnerability, capacity and exposure.^2^ The Hyogo framework for action has also already focused on child priority in the legislation program. ^3^ Accordingly, it is necessary to actively involve children in disaster risk reduction programs in order to overcome their needs and their problems. ^4^As children are more affected groups in various aspects of disasters in most countries, one should bear in mind their potential utilization and the conditions and space should be provided based on laws, national policies, training and capacity. Although after a disaster, children have particular needs and require attention, ^5,6^they should be considered as an active group who could participate in the DRR program and help their family and also the community.^4,7^

Some evidence suggests the value of children team working for community preparedness. Iran has had successful experience from using adolescent capacity as a pillar in activation of early warning; this has included providing notification for the local community when observing the rising sea levels in order to reduce the risk of flood disaster in a local area in the North of Iran.

According to the Hyogo and the Sendai documents, it seems that using the capacity of community, particularly with a focus on children in risk assessment, disaster risk reduction and disaster preparedness in communities are therefore more important than ever. To our best knowledge, due to the scarcity of studies in child capacity for disaster risk reduction, it is necessary that researchers concentrate on further studies regarding how to use children’s potential to reduce the risk of both natural and manmade disasters in forthcoming years.
